# Structural Accountability and Practice-Level Governance as Dual Pathways to Accreditation-Seeking Intention in Private Dental Practice: Evidence from a Mixed Public–Private Healthcare System

**DOI:** 10.3390/healthcare14131922

**Published:** 2026-07-01

**Authors:** Radu Ilinca, Laura Iosif, Dan Adrian Luțescu, Iulia Ioana Stănescu Spînu, Albert Zsolt Barabas, Ruxandra Ionela Sfeatcu, Ionela Ganea, Ana-Maria Cristina Ţâncu, Tudor-Claudiu Spînu

**Affiliations:** 1 Department of Medical Informatics and Biostatistics, Faculty of Dentistry, “Carol Davila” University of Medicine and Pharmacy, 4-6 Eforie St, 050037 Bucharest, Romania; radu.ilinca@umfcd.ro; 2 Department of Prosthodontics, Faculty of Dentistry, “Carol Davila” University of Medicine and Pharmacy, 37 Dionisie Lupu Street, District 2, 020021 Bucharest, Romania; laura.iosif@umfcd.ro (L.I.); anamaria.tancu@umfcd.ro (A.-M.C.Ţ.); tudor.spinu@umfcd.ro (T.-C.S.); 3 Discipline of Physiology, Faculty of Dentistry, “Carol Davila” University of Medicine and Pharmacy, 8 Eroilor Sanitari Bd, District 5, 050474 Bucharest, Romania; iulia.stanescu@umfcd.ro; 4 Department of Electrical Engineering and Information Technology, Faculty of Engineering, “G.E. Palade” University of Medicine, Pharmacy, Science and Technology, 1 Nicolae Iorga Street, 540088 Târgu-Mureș, Romania; 5 Oral Health and Community Dentistry Department, Faculty of Dentistry, Carol Davila University of Medicine and Pharmacy, 041292 Bucharest, Romania; ruxandra.sfeatcu@umfcd.ro; 6 3rd Modern Languages Department, Faculty of Medicine, “Carol Davila” University of Medicine and Pharmacy, 37 Dionisie Lupu Street, District 2, 020021 Bucharest, Romania; ionela.ganea@umfcd.ro

**Keywords:** accreditation-seeking intention, fee-for-service dentistry, quality governance, healthcare professionalism, financing of oral healthcare, dental public health

## Abstract

**Background/Objectives**: Private dental markets operating under fee-for-service arrangements lack the structural accountability mechanisms that typically drive quality governance adoption in publicly funded systems. In Romania, where no private dental office currently holds formal accreditation, accreditation-seeking reflects a genuine behavioral choice rather than a regulatory compliance. This study examined whether financing structure and Professional Governance behavior operate as distinct determinants of accreditation-seeking intention among private dental practitioners and which pathway offers a more tractable entry point for quality policy intervention. **Methods**: A cross-sectional survey was conducted among 98 licensed private dental practitioners in Romania (November 2025–January 2026). Three domain composites were derived from a 31-item de novo instrument anchored in JCI, ANMCS, and EN ISO 9001:2015 standards: Professional Governance, Patient Rights and Ethics, and Patient Safety Behaviors. The financing profile was operationalized as engagement with the national public insurance system (CNAS). Binary logistic regression modeled high accreditation-seeking intention (Q31 ≥ 4) against standardized domain scores and financing engagement, with bootstrap confidence intervals from 2000 replicates. **Results**: Two predictors independently and significantly predicted high accreditation-seeking intention. CNAS engagement showed the strongest association (OR = 5.05, 95% bootstrap CI [1.87–17.91], and *p* = 0.003), while the Professional Governance score remained independently associated (OR = 2.68, 95% bootstrap CI [1.63–5.63], and *p* = 0.003). The Patient Rights and Ethics score was not significant. Practitioners actively exploring CNAS contracting showed the highest accreditation-seeking intention (exceeding that of established contractors), suggesting a possible transitional accountability dynamic during financing transition, though this interpretation requires prospective longitudinal validation. Model performance was good (AUC = 0.788; Nagelkerke R^2^ = 0.339). **Conclusions**: Both financing engagement and governance behavior predicted accreditation-seeking intention after mutual adjustment, representing distinct pathways to voluntary quality adoption in private dental practice. Policy interventions aligned with entry into public contracting and with practice-level governance development are more likely to support accreditation uptake than normative appeals to professional ethics alone.

## 1. Introduction

Private dental markets occupy an unusual structural position within healthcare: they concentrate clinical care within commercially organized firms and expose that care to competitive market pressures. However, they simultaneously claim the ethical obligations and self-regulatory authority characteristic of learned professions [1,2], which leads to a tension that is not merely philosophical. Whenever services are financed almost entirely through fee-for-service out-of-pocket payments, practitioners face no payer-mediated accountability for population-level quality standards and bear no contractual obligation to adopt governance frameworks designed for publicly accountable providers [3,4]. External quality oversight in such systems tends to remain residual, voluntary, and weakly enforced, therefore placing the burden of quality assurance primarily on individual professional conduct rather than on structural incentives [5].

The dental profession has long attempted to address this imbalance through normative frameworks. Foundational ethical principles, as they are codified in the FDI Ethics in Dentistry policy statement, professional conduct standards, and international quality systems (such as Joint Commission International and EN ISO 9001) articulate commitments to patient welfare, beneficence, and accountability that are intended to operate independently of the financing structure [6,7]. Empirical research consistently confirms that dental practitioners broadly endorse these values, patient trust, professional responsibility, and accountability, which are widely recognized as core dimensions of dental professionalism across practitioner and public perspectives [8,9,10]. However, in practice, the alignment between endorsed professional values and structured governance behavior is uneven. Commercially oriented practice settings introduce incentive structures that operate independently of individual professional intention, particularly whenever revenue depends on procedural volume rather than on population outcomes [3,11]. Recent research on aesthetic dentistry has documented how market-driven pressures can erode the primacy of patient welfare even among practitioners who endorse professional values [12,13]. The result is a persistent and well-documented gap between widely endorsed ethical norms and the uneven implementation of governance practices across private dental systems.

Financing structures are a key structural determinant of this gap. Provider payment systems shape not only clinical behavior but also the accountability pressures which practitioners experience and the governance orientation they develop [3,4]. Fee-for-service remuneration, which dominates most European private dental markets, strongly incentivizes procedural activity while generating no structural pressure toward external certification, systematic documentation, or compliance with quality frameworks designed for publicly accountable providers [4]. Integration with public financing introduces a different logic: publicly contracted practitioners are subject to reporting requirements, payer scrutiny, and external accountability expectations that may promote governance-oriented behavior independently of intrinsic professional motivation [5,14]. The EU PRUDENT project has identified the predominance of fee-for-service as being among the key structural barriers to governance reform in European oral health systems, precisely because practitioners that operate within private-market logic bear no contractual accountability to quality standards designed for publicly funded care [4]. Addressing this requires understanding how transitions into public contracting alter the accountability environment and whether those transitions translate into measurable changes in quality-seeking behavior at the practitioner level.

The political economy literature suggests that accountability pressure is not constant across stages of financing integration. It may be most pronounced at the point of entry into public contracting before administrative demands become an institutionalized routine—a pattern that would be consistent with a transitional activation mechanism, though this conceptual possibility remains empirically untested at the practitioner level [5]. Despite this conceptual plausibility, whether financing engagement translates into measurable differences in accreditation-seeking intention at the practitioner level remains unexamined. Accreditation-seeking intention, which is a largely understudied outcome, links Professional Governance orientation to formal quality certification and captures a behavioral commitment that precedes and predicts actual adoption. The Lancet has called for the structural reform of treatment dominated by profit-driven dental systems as a prerequisite for meaningful quality improvement; the present study examines whether financing-level transitions may constitute a tractable near-term lever toward that goal [15].

This evidence gap is particularly consequential in Central and Eastern European contexts, where dental care is delivered predominantly through private markets with high out-of-pocket expenditure and limited public oversight infrastructure and also where the formal quality accreditation of private dental practices remains effectively absent [14,16]. Romania exemplifies this configuration. Dental care is provided overwhelmingly by privately operating practitioners, with only partial and voluntary integration into the publicly financed system managed by the National Health Insurance House (CNAS). Under Romanian law (Law 185/2017, Art. 7), private dental offices are explicitly exempted from the mandatory accreditation framework that applies to other ambulatory healthcare providers, meaning that no regulatory obligation generates structural pressure toward accreditation-seeking at the practice level. As a result, no private dental office in Romania currently holds accreditation from ANMCS or from international bodies such as JCI—a condition that removes both competitive visibility and peer benchmarks, while concentrating governance decisions entirely at the level of individual practitioners [17]. Under these conditions, accreditation-seeking intention is a genuine behavioral outcome rather than a regulatory compliance artifact, making Romania an analytically informative case for studying the determinants of voluntary quality adoption in a private dental system. Research in Romanian hospital settings has documented substantial variation in governance behavior and patient safety culture across provider types and organizational contexts, suggesting that analogous variation likely characterizes dental practice as well [18].

The existing empirical research has approached these dynamics primarily through normative or qualitative lenses by focusing on professional values, ethical tensions, and institutional design rather than by measuring governance behavior and its determinants directly [1,8]. Accreditation-seeking intention sits at the intersection of structural context and professional orientation: it reflects both the exposure to external accountability pressures and the internal governance practices of the dental office. Understanding which of these pathways exerts a greater influence on quality-seeking behavior has direct implications on how professional associations, policymakers, and accreditation bodies design interventions in order to expand voluntary governance adoption in private dental markets. Braithwaite et al.’s foundational conceptualization of accreditation as a socio-organizational process shaped jointly by external standards pressure and internal organizational culture provides the theoretical architecture for this dual-pathway inquiry [19], but neither pathway has been empirically quantified at the practitioner level in a private dental setting without mandatory accreditation requirements.

The present study contributes to the literature on voluntary quality adoption in mixed public–private healthcare systems. Accreditation-seeking intention is not merely a quality management outcome, but a behavioral expression of an organized profession’s self-regulatory commitment, linking individual providers to the institutional frameworks through which healthcare discharges its fiduciary obligations to patients and society. The FDI’s foundational framing of dentistry as a profession defined by beneficence, accountability, and patient welfare implies that the voluntary adoption of quality governance standards is an ethical act, not merely an administrative one [6]. However, whether this normative commitment translates into differentiated governance behavior and under what structural conditions it does so remains empirically unexamined at the practitioner level in private healthcare markets operating without mandatory accreditation requirements. This gap between ethical principles and measurable governance behavior is precisely the terrain which the present study maps.

This study addresses precisely this gap. By using cross-sectional survey data from 98 licensed private dental practitioners in Romania, a high out-of-pocket, predominantly fee-for-service market representative of the broader Central and Eastern European configuration, we examine how the financing profile and Professional Governance behavior independently predict accreditation-seeking intention, while accounting for Patient Rights and Ethics orientation. The objective is to determine whether structural financing incentives and internal governance orientation represent distinct and separately operating pathways to voluntary quality-seeking in private healthcare markets and to identify which pathway offers the most tractable entry point for policy and professional development interventions in mixed public–private systems.

## 2. Materials and Methods

### 2.1. Study Design and Setting

A cross-sectional survey was conducted among licensed dentists working in private dental offices in Romania between November 2025 and January 2026. The cross-sectional design was used to examine professional practice profiles, financing interface characteristics, and accreditation-seeking intention within a defined practitioner population at a single time point. Similar cross-sectional approaches have been widely used to study professional behavior and quality culture in dentistry [12,20,21]. The study was approved by the Ethics Committee of “Carol Davila” University of Medicine and Pharmacy, Bucharest (Protocol No. 33065/14.11.2025). The informed consent was obtained electronically from all participants prior to questionnaire completion. No personal identifiers were collected, and the participation was voluntary and anonymous.

Romanian dental care is delivered predominantly through private practices operating under fee-for-service arrangements, with selective integration into the publicly financed system managed by the National Health Insurance House (CNAS). This configuration mirrors the broader Central and Eastern European pattern characterized by high out-of-pocket expenditure, limited regulatory infrastructure, and heterogeneous quality assurance adoption [14,16]. The provider payment systems have been identified as structural drivers of governance adoption and quality-seeking behavior [3,4,22].

### 2.2. Participants and Sampling

The recruitment used purposive non-probability sampling through four electronic channels: national professional dental associations, scientific congresses including QR-code distribution at exhibition venues, professional online networks, and academic outreach through “Carol Davila” University of Medicine and Pharmacy in Bucharest. By taking into account that no comprehensive national registry of private dental practitioners exists in Romania, probability-based sampling is impracticable. Purposive sampling allowed for representation across practice sizes, geographic locations, and financing profiles central to the study hypotheses. Similar approaches are used in exploratory dental professionalism research [8,10].

Eligibility required active clinical practice within a private dental setting. No restrictions were placed on specialty, practice scale, or location. Data were collected on a dedicated online platform configured to require responses to all items before submission; consequently, all submitted questionnaires were fully completed, and no missing-data imputation was necessary. The final analytical sample comprised 98 respondents.

The practice configurations included single-unit offices (24.5%), 2–5-unit practices (43.9%), and networks exceeding five units (31.6%). Respondents were distributed across rural areas (2.0%), small towns (11.2%), county cities (36.7%), and university centers (50.0%). Organizational setting has been shown to shape governance behavior independently of individual attitudes in dentistry [1,2,20].

### 2.3. Survey Instrument

Data were collected via a structured self-administered questionnaire developed de novo for this study available in Supplementary Materials. No validated instrument for measuring professional practice profiles or accreditation-seeking intention in Romanian private dental practice was available. Instrument development followed approaches commonly used in exploratory implementation and accreditation research when no validated population-specific instrument is available [19,23]. A literature review identified no validated instrument measuring accreditation-seeking intention or Professional Governance orientation among private dental practitioners. The only related instrument identified, the Dental Patient Questionnaire [24], was designed to evaluate patient experience within an accreditation scheme rather than practitioner governance orientation, supporting the need for a de novo instrument in the present study. Item content was derived from three operational quality frameworks with direct relevance to Romanian dental practice: Joint Commission International standards [25], ANMCS standards [26], and EN ISO 9001:2015 [27]. These frameworks were selected because they represent the primary quality governance and certification pathways accessible to Romanian healthcare providers, with ISO 9001 certification relevant to public procurement eligibility and JCI accreditation increasingly pursued by private hospital networks operating in Romania. Item generation was performed iteratively by the authorship team, which includes licensed dental practitioners and university faculty with experience in quality governance, healthcare accreditation, and dental education. The review process focused on conceptual alignment with the source frameworks, clarity of wording, and relevance to the Romanian private dental practice environment. As part of scale refinement, item-total correlations were examined, and poorly discriminating items were removed prior to final analysis. The resulting instrument should be regarded as exploratory; formal psychometric validation, including external expert review, pilot testing, and confirmatory factor analysis in independent samples remains necessary before broader application.

### 2.4. Variable Construction

Outcome variable: Accreditation-seeking intention was assessed using item Q31 (“*How likely are you to seek accreditation for your dental office within the next three years?*”) rated on a five-point scale from 1 (very unlikely) to 5 (very likely). Single-item measures of behavioral intention are well established in implementation science research, where a single behaviorally anchored item with a defined time horizon frequently provides sufficient precision for predicting a concrete future behavior [28]. Q31 was designed to capture one specific behavioral commitment (the likelihood of seeking accreditation within a defined three-year horizon) rather than a multidimensional latent construct requiring triangulation across items. We acknowledge that accreditation-seeking intention may encompass additional dimensions such as organizational readiness, feasibility perceptions, and financial considerations. However, in the absence of a validated multi-item instrument for this construct in private dental practice, a single behaviorally anchored item represents reasonable and transparent operationalization for exploratory research in this domain. This approach is consistent with exploratory research practices in this field [19,28]. For logistic regression, responses ≥ 4 were classified as high accreditation-seeking intention, yielding 37 outcome events. This threshold was selected to identify practitioners with a clear positive intention, corresponding to “*likely*” or “*very likely*” on the response scale, while retaining sufficient events for stable regression estimation. We acknowledge that dichotomization results in some loss of information; accordingly, sensitivity analyses re-ran the model under a liberal threshold (Q31 ≥ 3; 68 events) and a strict threshold (Q31 = 5; 11 events). The ordinal logistic regression on the full five-point outcome was performed as a robustness check [28]. The consistency of findings across these specifications supports the robustness of the primary threshold choice.

Domain predictors: Domain composite scores were calculated as mean normalized item scores and rescaled to 0–100. For regression analyses, domain scores were standardized to z-scores (mean = 0, SD = 1), enabling direct comparison of odds ratios across predictors [29].

Financing predictor: Financing profile captured CNAS engagement using five categories: no contracts, private contracts only, no interest in CNAS, exploring CNAS, and active CNAS contractor. For the main regression, a binary variable grouped both exploring and active respondents as being engaged. This operationalization reflects the structural transition associated with entering public contracting [5]. A three-level ordinal specification (no engagement/exploring/active) was retained for robustness analyses.

### 2.5. Measurement Properties

Internal consistency was assessed using McDonald’s omega (ω) as the primary reliability coefficient, with Cronbach’s alpha (α) reported for comparability with existing dental survey literature. Omega is preferred for de novo multi-item scales where items have unequal factor loadings, a condition common in professional behavior composites drawing content from heterogeneous operational domains [30]. Partial Spearman correlations jointly controlling for age and professional experience were computed following the rank-based covariate-adjustment framework [31]. Bootstrapped 95% confidence intervals were derived from 2000 resampling replicates.

### 2.6. Statistical Analysis

All analyses were conducted in JASP (ver. 0.96 Apple Silicon, University of Amsterdam, Amsterdam, The Netherlands). Statistical significance was set at α = 0.05.

Descriptive statistics summarized continuous variables as mean ± SD, median, and interquartile range (IQR) and categorical variables as frequencies and percentages. Domain composite scores were computed on a 0–100 scale.

Spearman rank-order correlations evaluated associations between domain scores and accreditation-seeking intention (Q31, 1–5). Spearman was chosen in preference to Pearson, given the ordinal nature of all domain composites and the outcome variable and non-normal distributional characteristics of bounded Likert-type scores. Partial Spearman correlations jointly controlling for age and professional experience were computed due to strong collinearity between these variables [21]. Group differences across financing categories were assessed using the Kruskal–Wallis test with Epsilon-squared (ε^2^) as effect size [29]. Dunn post hoc comparisons used Benjamini–Hochberg correction [32].

Associations between binary accreditation intention and categorical predictors were examined using chi-square tests with Cramér’s v. Fisher’s exact test, which was applied when expected counts were small [10,33].

Binary logistic regression modeled high accreditation-seeking intention using three predictors. Continuous predictors were standardized to z-scores. The predictor count was constrained to three in accordance with the events-per-variable (EPV) criterion proposed by Peduzzi et al. [34]. With 37 outcome events and three predictors, the resulting EPV was 12.3, therefore exceeding the commonly cited minimum threshold of 10. Contemporary methodological literature has emphasized that EPV alone is insufficient for evaluating sample size adequacy in prediction modeling. Refs. [34,35] recommend considering total sample size, outcome proportion, and expected model performance jointly. In the present study, the total sample size (n = 98), outcome proportion (37.8%), and observed discrimination (AUC = 0.788) are consistent with factors identified as important determinants of prediction model performance. In addition, bootstrap confidence intervals based on 2000 replicates (seed = 42) were used to improve parameter stability and provide internal validation, which is consistent with current methodological recommendations for smaller datasets [36]. Multicollinearity among predictors was assessed using Variance Inflation Factor (VIF) and tolerance statistics. All VIF values were below 1.5 (Professional Governance: VIF = 1.470; Patient Rights and Ethics: VIF = 1.489; and CNAS engagement: VIF = 1.017), which indicates the absence of problematic multicollinearity. Corresponding tolerance values exceeded 0.67 for all predictors, well above the conventional threshold of 0.10. The model performance was evaluated using Nagelkerke R^2^, likelihood-ratio test, AUC, and the Hosmer–Lemeshow statistic.

Ordinal logistic regression using the full five-point outcome was performed as robustness analysis. Sensitivity analyses tested alternative thresholds and CNAS coding. Bootstrap confidence intervals were computed using 2000 replicates (seed = 42 for reproducibility). Multiple testing correction was applied using the Benjamini–Hochberg (BH) procedure across the primary family of hypothesis tests by covering Spearman correlations, group difference tests, and chi-square analyses. The BH correction was applied only to the bivariate analysis family; *p*-values from multivariable regression models are reported unadjusted. BH controls the false discovery rate under positive dependence, which is appropriate here, given the documented positive intercorrelations among domain scores [32]. The Benjamini–Yekutieli procedure, which controls FDR under arbitrary dependence, was applied as a confirmatory analysis. Analyses were pre-specified and limited to a small predictor set in order to avoid model overfitting.

## 3. Results

### 3.1. Sample Characteristics

A total of 98 respondents were included. Most of them were female (n = 67, 68.4%), with a mean age of 41.26 years (SD = 9.83) and mean professional experience of 16.05 years (SD = 9.78). Most of them practiced in 2–5-unit offices (43.9%) or networks exceeding five units (31.6%), while half the sample worked in university centers (50.0%). The financing profile is divided as follows: no CNAS interest (36.7%), no contracts (29.6%), active CNAS contract (17.3%), exploring CNAS (12.2%), and direct private contracts only (4.1%). Thirty-seven respondents (37.8%) reported “likely” or “very likely” accreditation-seeking intention (Q31 ≥ 4). Table 1 summarizes the sociodemographic and practice characteristics of the analytical sample, together with the distribution of the financing profile and accreditation-seeking intention.

### 3.2. Domain Reliability and Descriptive Scores

Domain composite scores on the 0–100 scale showed clearly differentiated levels. Patient Rights and Ethics scored highest and with the least spread (mean = 87.88, SD = 15.85; median = 93.75), followed by Patient Safety Behaviors (mean = 75.51, SD = 13.33; median = 80.00). The Professional Governance domain had both the lowest mean and greatest variability (mean = 61.15, SD = 18.31; median = 63.19). Mean accreditation-seeking intention was 2.97 on the original 1–5 scale (SD = 1.30; median = 3.00). Internal consistency, assessed using McDonald’s omega (ω) as the primary coefficient, was acceptable for Professional Governance (ω = 0.772, α = 0.654) as marginal but workable for Patient Rights and Ethics (ω = 0.705, α = 0.539). The Patient Safety domain showed low consistency (ω = 0.493, α = 0.274). This outcome is partly structural: item Q14 attracted the maximum response category from 95 out of 98 respondents (96.9%), therefore leaving negligible discriminant variance for hand hygiene compliance. Given this constraint, the Patient Safety Behavior domain scores carry a secondary and exploratory status throughout; all primary inferences rest on the governance domain and on the multivariable model.

### 3.3. Professional Governance and Accreditation-Seeking Intention

**Figure 1:**
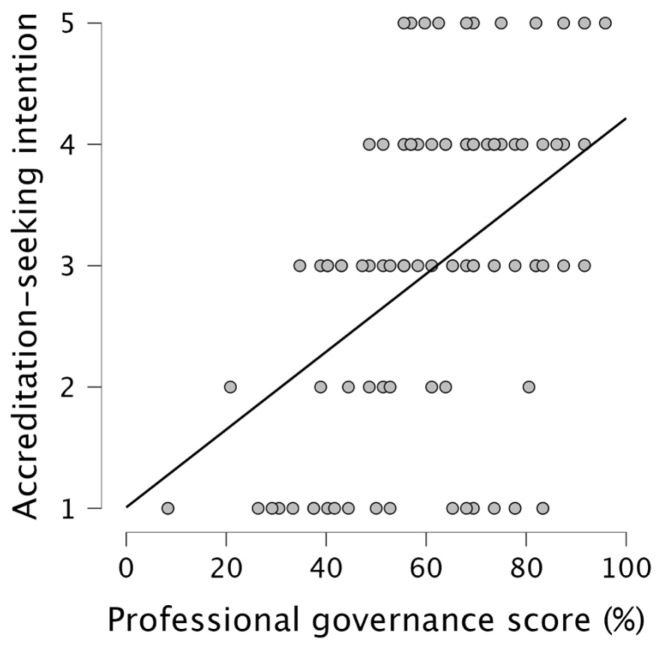
Scatter plot of Professional Governance composite score (0–100 scale) against accreditation-seeking intention (Q31, 1–5 scale) among private dental practitioners in Romania (n = 98). The fitted line represents the Spearman rank-order association (rs = 0.420, *p* < 0.001).

Figure 1 illustrates the association between the Professional Governance domain score and accreditation-seeking intention across the sample.

The Professional Governance domain showed a moderate, statistically significant positive association with accreditation-seeking intention (Spearman rs = 0.420, p_BH < 0.001; 95% bootstrap CI [0.223, 0.582]). The bootstrap interval, derived from 2000 resampling replicates, excluded zero and supports the stability of this estimate at N = 98. Neither the Patient Rights and Ethics domain (rs = 0.180, p_BH = 0.127) nor the Patient Safety domain in its primary capacity (rs = 0.263, p_BH = 0.022, treated as secondary and exploratory) matched this association in magnitude or consistency. Age and professional experience were not associated with accreditation-seeking intention (both rs < 0.07; p_BH > 0.60). Because age and experience were strongly correlated (rs = 0.982), both were treated jointly in a partial Spearman analysis. By controlling them simultaneously, the governance-accreditation relationship remained essentially unchanged (partial rs = 0.403, *p* < 0.001, and n = 98), which indicates that the association reflects professional orientation rather than career stage.

### 3.4. Financing Interface and Accreditation-Seeking Intention

**Figure 2:**
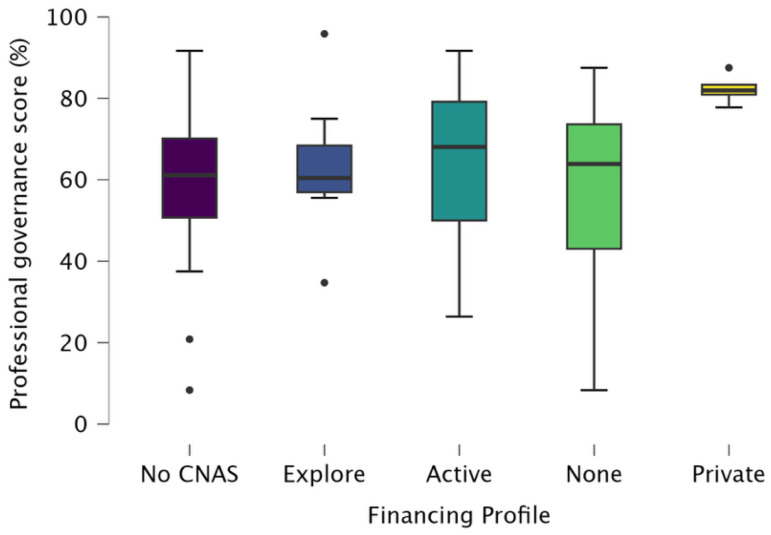
Distribution of accreditation-seeking intention (Q31, 1–5 scale) by financing profile category among private dental practitioners in Romania (n = 98). Groups: no contracts, private contracts only, no interest in CNAS, exploring CNAS, and active CNAS contractor. Kruskal–Wallis H (4) = 16.805, *p* = 0.002, and ε^2^ = 0.173. The dots represent individual outlier observations. The box indicates the interquartile range (IQR), the horizontal line within each box represents the median, and the whiskers extend to the most extreme observations within 1.5 × IQR. Points beyond the whiskers are plotted individually as outliers; no observations were excluded from the analysis.

Figure 2 displays the distribution of accreditation-seeking intention scores across the five financing profile categories.

Accreditation-seeking intention varied significantly across financing groups (Kruskal–Wallis H (4) = 16.805, *p* = 0.002, ε^2^ = 0.173, and large effect). The contrast was the sharpest for the exploring CNAS group, whose mean accreditation-seeking intention (4.33, SD = 0.78) considerably exceeded those of the no contracts (2.62) and no CNAS interest groups (2.78). The CNAS active group had a mean of 3.00, notably lower than exploring CNAS despite representing a more advanced stage of public contracting. Proportions reporting high accreditation-seeking intention (Q31 ≥ 4) were 83.3% among those actively exploring CNAS (10/12), compared with 47.1% for current CNAS contractors (8/17) and 27.5–30.6% across the remaining three groups. Chi-square analysis confirmed the association in the binary outcome (χ^2^(4) = 15.730, *p* = 0.003, Cramér’s V = 0.401, and moderate-to-large effect). Fisher’s exact test for exploring CNAS versus all other groups yielded OR = 10.926 (*p* = 0.001), indicating more than tenfold odds of high accreditation-seeking intention in this group alone. Dunn post hoc comparisons (Benjamini–Hochberg corrected) identified three significant pairwise contrasts: exploring CNAS vs. no contracts (p_BH = 0.001), exploring CNAS vs. no CNAS interest (p_BH = 0.001), and exploring CNAS vs. CNAS active (p_BH = 0.023).

### 3.5. Logistic Regression Model

Table 2 presents the binary logistic regression model predicting high accreditation-seeking intention, including odds ratios, bootstrap confidence intervals, and overall model fit statistics.

**Figure 3:**
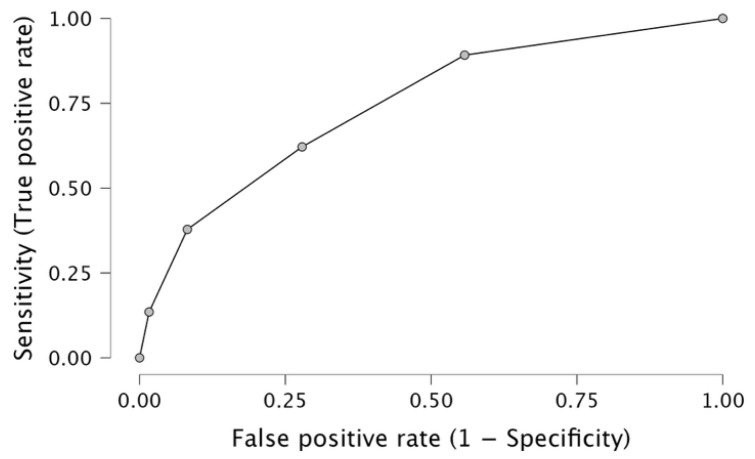
Receiver operating characteristic (ROC) curve for the binary logistic regression model predicting high accreditation-seeking intention (Q31 ≥ 4) among private dental practitioners in Romania (n = 98). The area under the curve (AUC) = 0.788 indicates good model discrimination. The diagonal reference line represents chance-level classification.

In order to evaluate the discriminative performance of the multivariable logistic regression model, a receiver operating characteristic (ROC) curve was constructed as seen in Figure 3.

**Figure 4:**
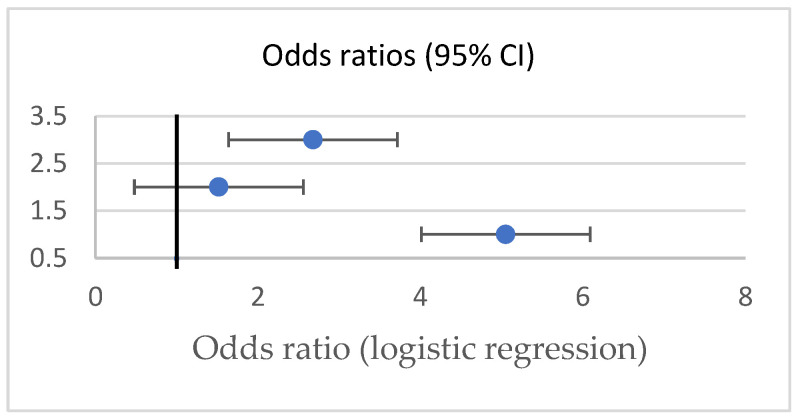
Forest plot of odds ratios (ORs) with 95% confidence intervals for predictors of high accreditation-seeking intention derived from the multivariable logistic regression model. The vertical reference line at OR = 1 indicates no association. Predictors with confidence intervals not crossing 1 are statistically significant.

In order to visualize the effect sizes and corresponding uncertainty of the predictors included in the logistic regression model, a forest plot of odds ratios with 95% confidence intervals was constructed, available in Figure 4.

Binary logistic regression identified two independent predictors of accreditation-seeking intention (Q31 ≥ 4). Each additional standard deviation in Professional Governance engagement was associated with approximately 2.7-fold higher odds of high accreditation-seeking intention (OR = 2.678, 95% bootstrap CI [1.628–5.634], and *p* = 0.003). CNAS engagement showed a larger effect: practitioners with any CNAS involvement had approximately five times the odds of high accreditation-seeking intention compared to those with no CNAS interface (OR = 5.051, 95% bootstrap CI [1.869–17.911], and *p* = 0.003). The Patient Rights and Ethics domain was not significant (OR = 1.520, 95% bootstrap CI [0.781–3.275], and *p* = 0.295). Overall model performance was good, with Nagelkerke R^2^ = 0.339, likelihood-ratio χ^2^(3) = 28.023, *p* < 0.001; AUC = 0.788; and Hosmer–Lemeshow χ^2^(8) = 8.087, *p* = 0.425, indicating adequate calibration.

### 3.6. Robustness Analyses

Robustness analyses were restricted to the Professional Governance and Patient Rights and Ethics domains; the Patient Safety Behaviors domain was excluded from all robustness specifications, consistent with its exploratory designation in Section 3.2, where low internal consistency and a severe ceiling effect on item Q14 precluded reliable composite interpretation. Findings involving this domain should therefore not be generalized beyond this sample.

Ordinal logistic regression on the full 1–5 Q31 response reproduced the binary model: both Professional Governance (OR = 2.302, 95% CI [1.444–3.671], and *p* < 0.001) and CNAS engagement (OR = 3.466, 95% CI [1.475–8.144], and *p* = 0.004) remained significant; the Rights and Ethics domain did not. Sensitivity analyses with alternative outcome thresholds confirmed directional stability throughout. Under the liberal threshold (Q31 ≥ 3, 68 events), governance remained significant (OR = 2.287, *p* = 0.008). Under the strict threshold (Q31 = 5, 11 events), CNAS engagement became dominant (OR = 8.361, *p* = 0.005), while governance approached but did not reach significance (OR = 2.456, *p* = 0.066). Replacing binary CNAS engagement with a three-level ordinal specification (no engagement/exploring/active) produced a significant per-step gradient (OR = 1.920, *p* = 0.034; AUC = 0.755), confirming that the finding is not an artifact of dichotomization. Bootstrap intervals from 2000 replicates were consistent with asymptotic estimates across all specifications.

## 4. Discussion

This study examined the structural and professional determinants of accreditation-seeking intention among private dental practitioners in Romania, which has a high out-of-pocket, predominantly fee-for-service market within the broader Central and Eastern European oral health policy landscape. The two independent predictors retained in the multivariable model, CNAS engagement (OR ≈ 5.1) and Professional Governance domain score (OR ≈ 2.7), align coherently with this sequence and invite interpretation against the existing literature on payment systems, dental professionalism, and quality governance. Taken together, these effects suggest that financing engagement exerts a stronger influence on accreditation orientation than on internal governance alone, while both of them operate as distinct and non-overlapping contributors.

Financing structure as a driver of accountability pressure: The dominant position of fee-for-service remuneration in Romanian private dentistry reflects a financing architecture that has been extensively characterized in the international literature as provider-incentive-intensive, cost-escalating, and weakly oriented toward population-level quality accountability [3]. Grytten’s analysis of dental remuneration systems identifies fee-for-service as the default arrangement that secures the procedural quality for performed treatments but creates no structural imperative for practitioners to seek external certification or submit to external governance frameworks [3]. The EU PRUDENT project has similarly highlighted that the predominance of fee-for-service in European oral health systems is among the key structural barriers to transformative governance reform, because practitioners operating entirely within private-market logic bear no contractual accountability to public payers and, consequently, face weaker institutional pressure to adopt quality standards designed for publicly accountable providers [4]. Romania’s oral health financing situation amplifies these dynamics: as documented by Winkelmann et al. and corroborated by recent scoping work on oral health coverage, European countries with the highest out-of-pocket expenditure shares consistently show the weakest integration between private dental providers and quality assurance infrastructure [14,16]. The present data extends this observation to the level of individual practitioner intention: practitioners who have no contractual interface with the public system show the lowest accreditation-seeking rates. Medico-legal pressures represent an additional and complementary source of accountability shaping practitioner behavior. Defensive medical practice, driven by fear of malpractice accusations and reputational risk, has been shown to induce both overuse and avoidance strategies, with systemic consequences including increased costs and reduced access to care [37].

The CNAS engagement effect and the activation phase hypothesis: The observation according to which practitioners who actively explore CNAS engagement showed higher accreditation-seeking intention than established contractors (mean 4.33 vs. 3.00) warrants careful contextualization. This pattern may be consistent with a transitional accountability dynamic: practitioners newly confronting the documentation and compliance demands of public contracting may show heightened quality-seeking orientation before those demands become routine and recede into the background. This interpretation is broadly consistent with the prior literature by suggesting that accountability pressures may be most salient when newly encountered rather than when they are fully institutionalized, although we treat this as a speculative hypothesis rather than a confirmed mechanism. Since cross-sectional design and small subgroup size (n = 12) preclude causal attribution, longitudinal data tracking practitioners through contracting transitions would be required to test this interpretation prospectively. The lower accreditation intention observed among established CNAS contractors may partly reflect post-adoption accommodation, although this interpretation equally requires longitudinal validation.

Professional Governance as an independent pathway: Beyond financing, Professional Governance engagement emerged as an independent predictor of accreditation-seeking intention, with an effect that was robust across model specifications and threshold sensitivity analyses. These finding positions governance orientation not merely as a correlate of compliance behavior but as a practitioner-level professional attribute that shapes quality-seeking independently of external financial incentives. This aligns with the dental professionalism literature’s persistent concern about the tension between professional and commercial imperatives in private dental practice. The dental professionalism literature has documented persistent tension between commercial practice environments and the fiduciary obligations of the dental profession by identifying governance orientation and structural accountability within the practice as the mechanisms through which professional values are operationalized rather than merely endorsed [1,8,11]. Practitioners with strong governance orientation in this sample appear consistent with the professional orientation identified in the dental professionalism literature as a counterweight to purely market-driven practice. Cserző et al. similarly found that structural governance mechanisms within the practice were seen as tangible expressions of professional accountability across practitioner and public perspectives [8]. The governance-accreditation pathway identified here suggests that this attitudinal–behavioral alignment has measurable downstream consequences for formal quality-seeking intention, even in settings where accreditation carries no current regulatory requirement.

The absence of a significant association between the Patient Rights and Ethics domain and accreditation-seeking intention is, on its surface, the finding which is most likely to be read as a limitation of the present study, particularly in a Special Issue oriented towards dental ethics. Rather, we argue that it represents a substantive empirical contribution to the literature on professionalism and quality governance. The distribution of Patient Rights and Ethics scores (mean 87.88, SD 15.85) indicates the near-universal endorsement of rights-based norms across the sample. If ethical commitment of this kind were sufficient to drive accreditation-seeking behavior, normative education alone would constitute an adequate policy lever. The present data suggest it does not.

This finding is consistent with a broader pattern documented in the professionalism literature: the gap between endorsed professional values and structured governance behavior is well recognized and persistent [1,2,11]. Holden et al. have characterized the tension between professional and commercial obligations in dentistry as one that operates independently of individual ethical commitment, precisely because market incentives shape behavior through structural pathways that normative values alone cannot override [2]. The present data operationalize this tension at the level of a measurable behavioral outcome: practitioners who endorse patient rights norms at ceiling levels do not differ from their peers on accreditation-seeking intention, while those who engage in structured governance practices do. This finding is consistent with the possibility that structural and organizational conditions play a more important role than attitudinal reinforcement in differentiating accreditation-seeking behavior.

This distinction carries direct implications for policy and professional development. The present findings suggest that interventions focused exclusively on strengthening ethical awareness or rights-based education may be insufficient to produce measurable gains in voluntary accreditation uptake in private dental markets, while structural interventions aligned with financing transitions and practice-level governance development appear more tractable. The FDI Ethics in Dentistry 2024 policy statement frames patient autonomy, beneficence, and non-maleficence as foundational professional commitments rather than distinguishing attributes [6]; the present findings are consistent with this framing and suggest that these commitments, precisely because they are foundational and near-universally endorsed, function as a professional floor rather than a behavioral differentiator. Ustrell-Torrent et al. similarly characterize ethical responsibilities toward the patient as embedded and non-negotiable components of dental professional identity [7]. The ethics domain in this study appears to be measuring adherence to that professional floor rather than a gradient of professional ambition—a finding that complicates normative assumptions embedded in much of the dental professionalism literature and that deserves further empirical investigation in other private healthcare contexts.

Governance behavior as the mediating mechanism: The co-occurrence of financing engagement and governance behavior as independent predictors, rather than the attenuation of one when the other is included, suggests that these represent distinct and complementary pathways to accreditation-seeking intention rather than proxies for a single underlying construct. This structure is consistent with implementation science frameworks that distinguish between external incentives—captured here through CNAS engagement—and internal readiness, operationalized through the governance domain score [23]. The Consolidated Framework for Implementation Research explicitly identifies outer setting pressures (including payer relationships and regulatory environments) and inner setting characteristics (including organizational culture and quality orientation) as separate and additive drivers of implementation readiness. Braithwaite et al.’s foundational conceptualization of accreditation research similarly argues that accreditation is best understood as a socio-organizational process shaped simultaneously by external standards pressures and internal organizational culture [19]. The present data provide exploratory evidence for this dual-pathway model at the practitioner level in a setting where no pathway has previously been empirically examined.

Contextual specificity and the Central and Eastern European pattern: The findings carry particular relevance for the broader Central and Eastern European context, where dental care is delivered predominantly through private markets with limited public oversight infrastructure and where formal quality accreditation of private dental offices remains effectively absent [14]. The political economy of oral health policy in comparable settings is characterized by inertia driven by private provider interests and incremental rather than systemic quality reform [5]. In this context, the finding that approximately 38% of practitioners report high accreditation-seeking intention identifies a segment that may represent early adopters for quality governance initiatives, whose pathway to adoption can inform targeted policy design. The Lancet’s oral health series has called for the structural reform of treatment-dominated, profit-driven dental systems [15]; the present findings suggest that provider-level financing interface changes may be a more tractable near-term lever than normative appeals to professional identity alone.

Limitations: Several limitations require acknowledgment. The purposive non-probability sample recruited through professional networks, scientific congresses, and academic outreach may introduce selection bias toward practitioners with greater engagement in professional development, quality improvement, or governance-related activities. This self-selection mechanism may systematically overrepresent practitioners already predisposed toward accreditation-seeking, potentially inflating the observed prevalence of high accreditation-seeking intention (37.8%) and attenuating contrasts between groups. The true prevalence of accreditation-seeking intention in the broader private dental workforce is likely lower than observed here, and effect size estimates should be interpreted with this in mind. This limitation is inherent to exploratory survey research conducted in the absence of a comprehensive national sampling frame for private dental practitioners and should be considered when interpreting the generalizability of the findings. The geographic distribution of respondents across practice locations is broadly consistent with the national distribution of dental practices, and recruitment through scientific congresses reached practitioners across a range of financing profiles and practice configurations. These factors do not eliminate selection bias but suggest that the sample is not systematically restricted to a narrow segment of the practitioner population. The low proportion of rural respondents (2.0%) reflects the well-documented geographic maldistribution of dental practitioners in Romania, where only 10.8% of licensed dentists currently practice in rural areas [38]. Nevertheless, the limited representation of rural practitioners should be considered when interpreting the external validity of the findings. A further limitation concerns the possibility of common method bias. All principal study variables, including the domain composite scores and the accreditation-seeking intention measure, were collected simultaneously through the same self-administered survey instrument. Consequently, some observed associations may be partially inflated by shared method variance rather than reflecting entirely independent constructs, a limitation inherent to cross-sectional self-report research [19,23]. Although the financing variable was derived from a categorical practice characteristic rather than from a multi-item attitudinal scale, the possibility of common method bias cannot be excluded and should be considered when interpreting the magnitude of the reported associations. The cross-sectional design precludes causal inference; the activation phase interpretation of the exploring CNAS group, while theoretically coherent, requires longitudinal validation. The exploring CNAS group comprised 12 respondents; all pairwise contrasts and effect size estimates involving this subgroup, including Fisher’s exact OR of 10.926, should be interpreted with caution despite statistical significance, and replication in larger samples is necessary before policy recommendations are drawn specifically from this subgroup’s behavior. Certain financing categories were represented by very small numbers of respondents (e.g., private contracts only, n = 4), and findings involving these subgroups should therefore be interpreted cautiously. Accreditation intention is a self-reported proxy, and the gap between stated intention and actual behavior may be substantial in a context where no private dental office currently holds accreditation, removing demonstrated role models. Future research should test whether governance behavior mediates the financing–intention relationship by using structural equation modeling in larger samples and should track practitioners through CNAS contracting transitions to test the activation phase hypothesis prospectively.

The de novo instrument carries inherent validation limitations. No published psychometric validation history exists for this scale, and the present study cannot establish test–retest reliability, confirmatory factor structure, or convergent validity against established dental governance measures. No exploratory or confirmatory factor analysis was performed; consequently, the dimensional structure of the instrument remains preliminary, and the domain groupings reflect theoretically motivated content categories rather than empirically confirmed latent factors. Reliability coefficients alone are insufficient to establish construct validity, and this limitation should be kept in mind when interpreting domain-level findings. The Patient Safety Behaviors domain showed particularly low internal consistency (ω = 0.493; α = 0.274), partly attributable to a severe ceiling effect on item Q14, which attracted the maximum response from 95 out of 98 respondents, leaving negligible discriminant variance. For this reason, the Patient Safety domain was designated as exploratory and excluded from all primary inferences and robustness specifications; all substantive conclusions rest on the Professional Governance domain and the multivariable model. Formal validation in independent samples, including independent expert review of item content, a formal pilot study with cognitive interviewing, exploratory and confirmatory factor analysis, and criterion-related validity testing, is a necessary next step before the instrument can be recommended for broader use. These limitations should be considered when interpreting the domain-level findings.

Implications: Despite these limitations, the findings identify a practical entry point for policy and professional development. Quality initiatives aligned with the transition into public financing—particularly at the stage of CNAS engagement—may have a higher likelihood of uptake than general appeals to professional values or abstract accreditation mandates. Strengthening governance capacity within practices may independently support accreditation readiness for practitioners who remain outside the public system. Together, these pathways suggest that quality adoption in private dental systems is shaped not by professional commitment alone but by the interaction between structural context and organizational practice—a distinction that may inform the design of targeted, incentive-aligned quality policies in mixed public–private dental systems.

## 5. Conclusions

Accreditation-seeking intention in private healthcare markets is shaped by two distinct mechanisms: the structural accountability generated by engagement with public financing and the internal governance orientation cultivated within the practice. These pathways operate independently and require separate policy attention. Private dental practice, operating without mandatory accreditation requirements in a high out-of-pocket financing environment, provides an analytically tractable case for examining these mechanisms in their purest form.

Among all financing groups, practitioners actively exploring public contracting reported the highest accreditation-seeking intention, surpassing even established contractors. This pattern is consistent with a transitional accountability dynamic; however, given the cross-sectional design and the small subgroup size (n = 12), this should be regarded as a hypothesis requiring prospective longitudinal validation rather than an established finding. At the same time, governance behavior within the practice independently predicted accreditation orientation by indicating a parallel quality pathway available to practitioners operating entirely outside public financing.

For policy in mixed public–private healthcare systems, these findings identify two tractable intervention points: the financing transition, where accreditation support can be embedded within public contracting processes; and practice-level governance development, which can strengthen quality-seeking readiness irrespective of financing status. Normative commitment to patient ethics, while foundational to professional identity across healthcare, did not differentiate practitioners on this behavioral outcome; this is a finding consistent with the near-universal endorsement of rights-based values across the sample, which suggests that systemic quality expansion depends primarily on structural and organizational levers rather than on normative appeals alone.

Expanding accreditation uptake in private markets also carries equity implications: shifting quality assurance from individual ethical discretion toward verifiable structural standards may reduce systematic variation in care quality across patient income groups. In settings where voluntary adoption represents the only available pathway for governance development, the understanding of its structural and professional determinants is essential for designing realistic and context-specific strategies to support uptake. These findings are relevant beyond dentistry: Any private healthcare sector operating under fee-for-service arrangements without mandatory accreditation requirements faces structurally analogous conditions, and the dual-pathway model identified here may inform quality policy design across such contexts.

## Figures and Tables

**Table 1:** Sample characteristics of private dental practitioners in Romania (n = 98).

Variable	Category	n	%
Gender *	Female	67	68.4
	Male	31	31.6
Practice scale	Single-unit office	24	24.5
	2–5-unit office	43	43.9
	Network > 5 units	31	31.6
Location	Rural	2	2.0
	Small town	11	11.2
	County city	36	36.7
	University center	49	50.0
Financing profile	No contracts	29	29.6
	Private contracts	4	4.1
	No interest in CNAS	36	36.7
	Exploring CNAS	12	12.2
	CNAS active	17	17.3
Accreditation intention	Unlikely/neutral (Q31 1–3)	61	62.2
	Likely/very likely (Q31 ≥ 4)	37	37.8

* Age (years), mean ± SD: 41.26 ± 9.83; professional experience (years), mean ± SD: 16.05 ± 9.78.

**Table 2:** Binary logistic regression predicting high accreditation-seeking intention (Q31 ≥ 4) among private dental practitioners in Romania (n = 98).

Predictor	Odds Ratio (OR)	95% CI	*p*-Value
Professional governance	2.678	1.628–5.634	0.0028
Patient rights & ethics	1.520	0.781–3.275	0.2948
CNAS engaged	5.051	1.869–17.911	0.0026
Model performance	Nagelkerke R^2^ = 0.339, AUC = 0.788 Hosmer–Lemeshow χ^2^(8) = 8.09, *p* = 0.425		
	Likelihood-ratio test χ^2^(3) = 28.02, *p* < 0.001		

Note: Bootstrap 95% CI computed from 2000 replicates (seed = 42). p_BH = Benjamini–Hochberg corrected; key findings [BY] also significant under Benjamini–Yekutieli correction. The *p*-values shown in Table 2 are from the logistic regression model and are not subject to BH correction, which applies to the bivariate analysis family only.

## Data Availability

The data presented in this study is available upon reasonable request from the corresponding authors.

## Supplementary Materials

The following supporting information can be downloaded at: https://www.mdpi.com/article/10.3390/healthcare14131922/s1, The Questionnaire: QT1.

## Abbreviations

The following abbreviations are used in this manuscript:

ANMCS	National Authority for Quality Management in Healthcare (Romania)
AUC	Area Under the Curve (Receiver Operating Characteristic)
BH	Benjamini-Hochberg Procedure
BY	Benjamini-Yekutieli Procedure
CI	Confidence Interval
CNAS	National Health Insurance House (Casa Națională de Asigurări de Sănătate, Romania)
EPV	Events Per Variable
FDI	World Dental Federation
FDR	False Discovery Rate
IQR	Interquartile Range
ISO	International Organization for Standardization
ISQUA	The International Society for Quality in Health Care
JCI	Joint Commission International
OR	Odds Ratio
Q31	Questionnaire Item Measuring Accreditation-Seeking Intention
ROC	Receiver Operating Characteristic
SD	Standard Deviation
ω (Omega)	McDonald’s Omega Reliability Coefficient
α (Alpha)	Cronbach’s Alpha Reliability Coefficient
ε^2^	Epsilon-Squared (Effect Size for Kruskal-Wallis Test)
rs	Spearman’s Rank Correlation Coefficient
