# Supplementary material for: Structural Accountability and Practice-Level Governance as Dual Pathways to Accreditation-Seeking Intention in Private Dental Practice: Evidence from a Mixed Public–Private Healthcare System
Source: Healthcare (Basel). 2026 Jul 1;14(13):1922. doi: 10.3390/healthcare14131922 (PMC13361069; doi:10.3390/healthcare14131922)
Supplement: Supplementary file 1 [file healthcare-14-01922-s001.zip › healthcare-4351465-supplementary.pdf]

# Dentists' perception on the voluntary accreditation of dental services in Romania

## Survey Questionnaire

General information

Q1. My age is ........ years

Q2. My gender is:

- Male
- Female

Q3. I work:

- in my own office with only 1 unit
- in my own office with 2 - 5 units
- in a network of offices with over 5 units

Q4. I practice:

- in a university center
- in a county seat city
- in a small town
- in the countryside

Q5. I have …… years of professional experience.

Q6. How important is PATIENT RETENTION in maintaining and improving the reputation of your practice on a 1 to 5 scale, where 1 = Not important, 2 = Slightly important, 3 = So-so, 4 = Very important, 5 = Extremely important?

- 1 Not important
- 2 Slightly important
- 3 So-so
- 4 Very important

- 5 Extremely important

Q7. How important are INFORMAL PATIENT RECOMMENDATIONS (word-of-mouth) for maintaining and increasing the reputation of your practice on a scale from 1 = Not important, 2 = Slightly important, 3 = So-so, 4 = Very important, 5 = Extremely important?

- 1 Not important
- 2 Slightly important
- 3 So-so
- 4 Very important
- 5 Extremely important

Q8. How important are DIRECT FEEDBACK AND PUBLIC REVIEWS for maintaining and increasing the reputation of your practice on a scale from 1 = Not important, 2 = Slightly important, 3 = So-so, 4 = Very important, 5 = Extremely important?

- 1 Not important
- 2 Slightly important
- 3 So-so
- 4 Very important
- 5 Extremely important

Q9. How important are ACCREDITATION CERTIFICATES AND QUALITY ASSURANCE CERTIFICATIONS for maintaining and increasing the reputation of your practice on a scale from 1 = Not important, 2 = Slightly important, 3 = So-so, 4 = Very important, 5 = Extremely important?

- 1 Not important
- 2 Slightly important
- 3 So-so
- 4 Very important
- 5 Extremely important

Patient safety objectives

Q10. The clinic/office where I work:

- is in contractual relations with the National Health Insurance House

- currently does not have a contract with the National Health Insurance House, but is considering this option within the next 3 years
- is not interested in a contract with the National Health Insurance House and does not plan to pursue one in the next 3 years
- has direct contracts with private companies
- does not have any kind of contracts

Q11. How is the patient's identity currently verified before treatment?

- By asking for the patient’s name and by checking the written records
- Just by asking for the name
- By visual recognition
- By assuming the patient’s identity
- Without any verification

Q12. How are errors prevented in the administration of various substances used in your dental practice?

- Labels are double-checked
- The patient is asked about allergies
- Individual medications are used
- Personal professional judgment is used
- No specific method

Q13. How would you assess your systematic approach to preventing patient falls/injuries?

- No systematic approach – I rely only on general awareness
- Basic precautions – I take some safety measures, but not systematically
- Moderate Approach – I have consistent safety practices for most situations
- Good systematic approach – I follow the usual safety protocols, with occasional lapses
- Excellent systematic approach – I have comprehensive and consistent safety protocols for all situations

Patients' Rights and Communication

Q14. How do you ensure compliance with hand hygiene?

- Always before and after patients

- Usually
- Sometimes
- Rarely
- No specific routine

Q15. How do you inform patients about the risks of treatment?

- Detailed explanation
- Brief mention
- Only if requested
- Assumed understanding
- No discussion

Q16. How do you obtain patient consent for treatments applied?

- Written form
- Verbal agreement
- Implied consent
- No formal process

Q17. Rate how well you respect the patient's privacy during treatment.

- Poor – Privacy is not a primary concern during treatment
- Fair – I provide basic privacy, but with some gaps or inconsistencies
- Good – I generally ensure patient privacy, with occasional omissions
- Very good – I consistently maintain patient privacy, with minor exceptions
- Excellent – I always ensure complete patient privacy

Q18. How do you handle patient complaints?

- Immediate response
- Discuss later
- Refer to a colleague
- Defensive response
- Ignorance

Leadership and quality management

Q19. How do you demonstrate Leadership in Quality Assurance in your practice?

- Set clear standards
- Lead by example
- Delegate to staff
- Assume quality as default
- No specific approach

Q20. Rate your commitment to creating a patient safety culture.

- I am not employed – Patient safety culture is not a priority for my practice
- I consider patient safety culture, but it is not a central point of interest
- Moderately Committed – I am somewhat committed to improving the patient safety culture
- Highly committed – Patient safety culture is important and I'm actively working on it
- Extremely committed – Creating a safety culture is an absolute priority in everything I do

Q21. How often do you review practice performance data?

- Weekly
- Monthly
- Quarterly
- Annually
- Never

Q22. How do you ensure staff accountability for quality assurance?

- Regular feedback
- Performance evaluations
- Observation only
- Trust-based
- No specific method

Infection prevention and control

Q23. Rate your compliance with infection control protocols in medical practice:

- Poor compliance – I follow infection control measures inconsistently or minimally

- Appropriate compliance – I follow basic infection control methods, but with frequent gaps
- Good compliance – I generally follow infection control measures, with occasional instances of non-compliance
- Very good compliance – I consistently follow infection control measures, with rare exceptions
- Excellent Compliance – I always strictly adhere to comprehensive infection control measures

Q24. How do you handle contaminated materials?

- Strict protocols
- Careful handling
- Standard disposal
- Basic removal
- No specific method

Continuous improvement and process management

Q25. How do you identify areas for practice improvement?

- Patient feedback
- Self-assessment
- Colleagues' contribution
- Problem-oriented
- No systematic approach

Q26. Evaluate your use of data to make practical decisions:

- I never use data – I make decisions based solely on intuition and experience
- I rarely use data – I occasionally consider some information, but I mostly rely on experience
- I sometimes use data – I use the information available for some decisions, but not systematically
- I often use data – I regularly seek and use information to guide most practice decisions
- I always use data – I consistently base all my practice decisions on available data and evidence

Q27. How often do you implement changes based on patient satisfaction?

- Ever
- Often
- Sometimes
- Rare
- Never

Preparation and attitudes for implementation

Q28. Rate your willingness to implement written patient safety protocols:

- Not ready – I am not ready to implement written patient safety protocols at this time
- Somewhat ready – I have minimal readiness, but significant barriers remain
- Moderately ready – I am somewhat prepared, but I need more resources/time
- Very ready – I am well prepared and could implement protocols with minor adjustments
- Fully ready – I am fully prepared and could immediately implement written protocols

Q29. How important is international accreditation (such as Joint Commission International) for the reputation of your practice?

- Not important at all – International accreditation has no value to the reputation of my practice
- Slightly important – International accreditation could offer minimal reputational benefits
- Moderately important – International accreditation could provide some reputational benefits
- Very important – International accreditation would significantly improve the reputation of my practice
- Extremely important – International accreditation is essential for establishing a solid reputation in practice

Q30. Rate your willingness to adopt systematic quality measurement:

- I am not willing – I am not interested in adopting systematic quality measurement
- Somewhat willing – I have minimal interest, but significant reservations about systematic measurement
- Moderately willing – I am somewhat open to systematic quality measurement with appropriate support

- Very willing – I am eager to adopt systematic approaches to quality measurement
- Extremely willing – I am highly motivated and committed to implementing systematic quality measurement

Q31. How likely are you to apply for/ seek accreditation in the next 3 years?

- Very likely
- Probable
- Neutral
- Unlikely
- Very unlikely
